# Influence of Emojis on Online Trust Among College Students

**DOI:** 10.3389/fpsyg.2021.747925

**Published:** 2021-11-01

**Authors:** Mei Zhang, Shuheng Ding, Yining Liu, Hailong Li, Yanchun Zhu, Chunlei Qin

**Affiliations:** ^1^School of Sociology and Psychology, Central University of Finance and Economics, Beijing, China; ^2^Business School, Beijing Normal University, Beijing, China; ^3^Office of Human Resources, Central University of Finance and Economics, Beijing, China

**Keywords:** online trust, initial trust, interpersonal trust, emojis, college students, emotion, experiment

## Abstract

Emojis are increasingly used in online communication and expression, however, most previous studies have focused on describing this phenomenon, but less on how it affects interpersonal trust relationships. Therefore, this study examines the effect of emojis on online interpersonal trust among college students through three experiments. A total of 62 college students were recruited for Experiment 1. The results demonstrated that positive emoji (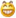) improved the level of trust of trustors in the trust game [*t*(60) = –2.79, *p* = 0.007], whereas that of the control group exerted no effect on the initial level of online trust among college students. Then, 74 college students were selected for Experiment 2. The results indicated no significant differences between the experiment and control groups in terms of the influence of negative emojis (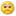) on initial online trust using. A joint analysis (*via* ANOVA) of Experiments 1 and 2 illustrated that the type of emoji exerted a significant effect [*F*(2,96) = 3.96, *p* = 0.02, η*^2^* = 0.08] on college students’ online trust. Finally, we recruited 111 participants for Experiment 3 to explore the role of emojis on online trust among acquaintances. The results suggested that the individual propensity to trust plays a moderate role in the relationship between emojis and online trust among acquaintances. That is, emojis influenced interpersonal trust among acquaintances only if the level of propensity to trust, is low.

## Introduction

Online social media frequently use specialized languages, such as abbreviations, hashtags, emoticons, and emojis as substitutes to compensate for the absence of non-verbal cues (Fathiya, 2018; Fischer and Herbert, 2021). Generally speaking, an emoji is a small digital image or icon used to express an idea or emotion (Ganster et al., 2012; Singh et al., 2019). With the development of information technology, especially smart phones, the concise and interesting features of emojis [smiley emoji (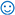) in particular] are increasingly used in computer-mediated communication (Gesselman et al., 2019; Beattie et al., 2020; Jones et al., 2020; Sweeney, 2020). At the same time, emojis from various manufacturers can communicate with one another through mail, web pages, mobile phones, and computer operating systems without hindrance, because their code adheres to the Unicode International Standard (Rodrigues et al., 2018). According to news reports in the United States, 74% of Americans use emojis to express feelings and emotions and an average of 96 emojis in text communication and on social media on a daily basis (Willoughby and Liu, 2018).

With the development of social networks, many changes have occurred in the social behaviors and interpersonal spaces of college students (Kahraman et al., 2020; Veytia-Bucheli et al., 2020). Teenagers typically use emojis on social networks as visual symbols that combine semiotics and human facial expressions to convey emotions and imply certain psychological states (Choi and Aizawa, 2019). Therefore, as important non-verbal symbols and new pictographic forms on social networks, emojis and their use imply important consequences for the social behaviors, social interactions, and even individual personality traits of college students (Marengo et al., 2017; Boutet et al., 2021; Was and Hamrick, 2021).

In social interactions, people are frequently required to decide whether to trust others. In studies on online trust, researchers generally adopt a definition of trust from the offline context. Interpersonal trust is a generalized expectation that the verbal statements of others are reliable (Rotter, 1967). Conversely, initial trust denotes a decision-making process in which a trustor quickly decides whether to exhibit an altruistic behavior with a trustee, which is based on the impression and experiences obtained from their first communication (McKnight et al., 2002; Shareef et al., 2020). The latter is a heuristic cognition and is more susceptible to trust clues and emotions (Couch and Jones, 1997). Previous experimental studies confirm that subtle social clues, especially those involving the human eyes, such as robots with large eyes or three regular triangular dots that mimic the human face, influence the trust levels of participants (Xin et al., 2016). Many expressions in emojis are represented by “small yellow round faces,” such as 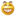 and 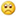. These faces not only contain clues about facial features, such as the eyes, but also convey emotional information through the details of the eyes, mouth, and other parts of the face. Therefore, the study infers that emojis are likely to influence the levels of trust in social interactions. Moreover, they directly convert expressions in the said text into an emotional symbol, which, thus, shortens the psychological distance between users (Jones et al., 2020). For college students, emojis provide an easy way to add personality to text-based communication. Moreover, sending emojis is much quicker than typing a response (Willoughby and Liu, 2018; Kabir and Marlow, 2019). Thus, using emojis not only narrows the psychological distance between users but also increases the likelihood of the said users to trust one another. In this regard, we present the following hypothesis:

***H1*** *: Emojis exert a significant positive predictive effect on initial online trust among college students.*

Given that emojis convey various forms of emotion-related information, such as *happiness*, *sadness*, and *fear*, the positive and negative emotions aroused and conveyed by these expressions may provide participants with different psychological cues. As a consequence, different emojis may also exert varying effects on interpersonal trust. According to the research on computer-mediated communication, cues from online virtual avatars can provide an accurate perception of extroversion and agreeableness of personality traits (Kim et al., 2012; Fong and Mar, 2015; Aseeri and Interrante, 2021). Compared with other clues, virtual images without expression can negatively influence initial online trust among investors. Moreover, many studies proved that emotions with positive and negative valences increase and decrease the levels of trust, respectively (Dunn and Schweitzer, 2005). Accordingly, the study proposed the following hypothesis:

***H2*** *: Emojis that express different emotions exhibit varying effects on initial online trust among college students.*

Moreover, previous studies found that emotions exert no effect on trust when a trustor and trustee are familiar with each other (Barbalet, 1996; Dunn and Schweitzer, 2005). How, then, does the relationship between emojis and trust influence acquaintances? What other variables interfere with the relationship between emojis and trust among online acquaintances? These issues have received little attention in previous studies.

In the real world, no initial trust exists among acquaintances, that is, people cultivate stable trusting relationships through blood ties, geography, and interpersonal interactions. In essence, trust among acquaintances within society is characterized by emotional trust (Welch et al., 2007). From this perspective, emojis as objective variables do not influence interpersonal trust among acquaintances. However, online trust is an extension of interpersonal trust in the cyber world, which is fragile and cognitive at the same time. On the one hand, individuals are frequently exposed to the risk of uncertainty, which leads to the slow process of constructing online trust without interruption or damage due to the anonymity and asynchrony of online communication (Shankar et al., 2002; Wilson et al., 2006). On the other hand, online communication produces more controversies than face-to-face communication due to the lack of non-verbal information and visual and auditory information (Joinson et al., 2010). As such, trustors and trustees pay more attention to a given task instead of the emotion, opinion, mood, or other social information that the said task may convey. As a useful supplement for non-verbal and emotional clues, emojis may also help in developing online trust among acquaintances.

In addition to online activity, individual propensity to trust may plays an important role in the relationship between emojis and online trust. The propensity to trust is usual means a consistent tendency to be willing to depend on others across a broad spectrum of situations and persons (McKnight et al., 1998). Such a propensity reflects one’s faith in humanity and capacity to trust others based on lifelong experiences and various forms of socialization (Gefen, 2000; Lewicki et al., 2006; Wu et al., 2010). Therefore, the study infers that if a trustor’s propensity to trust is high, then emojis as external clues will exert no effect on the interaction between trustees and trustors. However, if this propensity is low, then online interactions among acquaintances will be similar to initial online trust between strangers. Against this background, we propose that:

***H3*** *: Propensity to trust plays a moderating role in the relationship between emojis and online trust among acquaintances.*

In summary, this study proposes three hypotheses and designs three experiments to investigate the influence of emojis on online trust among college students. Experiments 1 and 2 are intended to verify the influence of emojis in expressing positive and negative emotions in relation to initial online trust between strangers. Moreover, Experiment 1 and 2 verified H1, and the joint analysis verified H2, respectively. Lastly, Experiment 3 investigates the effect of emojis on online trust among acquaintances as well as the role of individual propensity to trust in this relationship and verified H3. Given that previous studies have found that smiling smilies have a stronger impact on personal mood than smiling emoticons (Ganster et al., 2012), 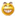 and 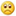 always appear in pairs and as representatives of positive and negative emojis (Novak et al., 2015; Was and Hamrick, 2021), these two emoji were chosen as the experimental materials for this study.

## Experiment 1: Influence of Positive Emojis on Initial Online Trust Among College Students

### Participants

We randomly selected a total of 62 college students (31 males, 31 females) in Beijing (average age = 21.55 years, *SD* = 1.48). According to the previous research (Miao et al., 2021), a sensitivity power analysis of the independent sample *t*-test in experiment 1 was conducted (assuming *α* = 0.05, power = 0.8) in G^∗^Power 3.1. The results turned out that the effect size *d* we calculated based on our sample size was 0.72, which was at the medium level (0.50 < *d* < 0.80). All participants were randomly assigned to experiment and control groups with 31 participants per group.

### Experimental Material

#### Positive Emoji

Based on previous pre-interviews and studies on emojis commonly used to express positive and negative emotions (Wahyuni and Budi, 2018), we selected a smiley emoji with an open smile and smiling eyes (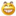), which is most commonly used in online chats to express positive emotions.

#### Initial Online Trust

**FIGURE 1:**
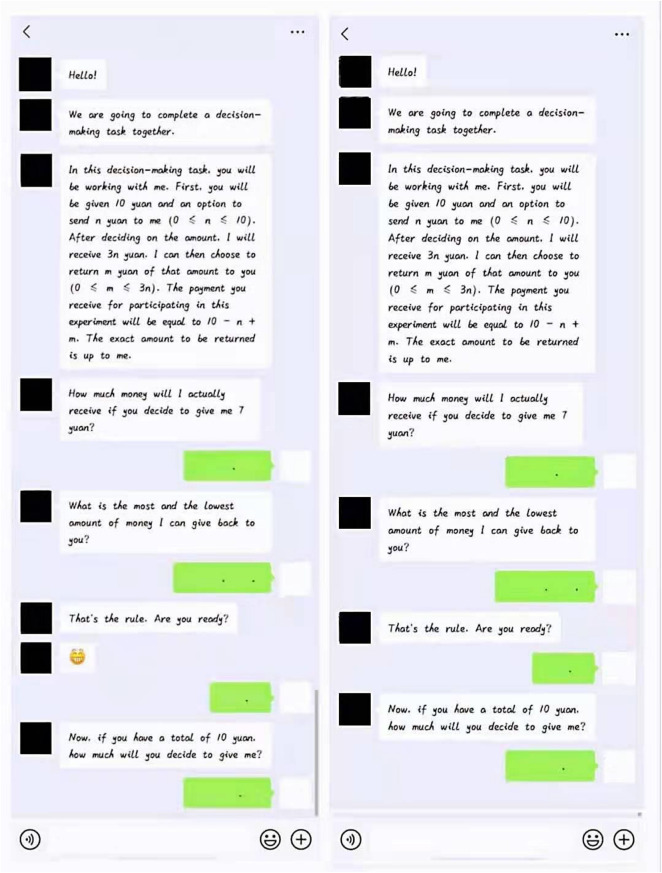
Two groups of college students playing the trust game in a WeChat dialogue box.

Similar to the classic paradigm posited by Berg et al. (1995), in the experiment, two players (i.e., a trustor and a trustee) were set up with a specific amount of money S (S = 10). The trustor was required to give a portion of money n (0 ≦ n ≦ S) to the trustee. Then, the trustee obtained 3n amount of money and decided to return the amount m (0 ≦ m ≦ 3n) to the trustor. Finally, the trustor finally earned S – n + m and the trustee earned S + 3n – m. We adapted the above tasks into WeChat version. According to the conclusion of previous studies, which suggest that emojis mainly appear at the end of sentences and in the lower two-thirds of tweets (Novak et al., 2015), this experiment situated the emojis after the explanation of the game rules (Figure 1).

### Experimental Design and Procedure

The experiment adopted a single-factor (emoji: expressionless/smiley) inter-participant design. The dependent variable was initial online trust, whereas operation was defined as the amount of money that the participant submitted. The monetary unit is Chinese yuan.

During the experiment, a trustor engaged in a dialog with a trustee in the simulated WeChat dialog box (Figure 1). The trustor was randomly manipulated into a conversation with an entirely unfamiliar Classmate A (the trustee, actually an experiment assistant). After greeting the trustor, Classmate A invites he/her to participate in the experiment (“We are going to complete a decision-making task together”) and then sent a message to introduce the rules of the trust game, then posed questions to verify whether the participants understood the rules. The game rule is presented as follows: “In this decision-making task, you will be working with me. First, you will be given 10 yuan and an option to send n yuan to me (0 ≤ n ≤ 10). After deciding on the amount, I will receive 3n yuan. I can then choose to return m yuan of that amount to you (0 ≤ m ≤ 3n). The payment you receive for participating in this experiment will be equal to 10 – n + m. The exact amount to be returned is up to me.” Then, all participants completed the following manipulation test question: “How much money will I actually receive if you decide to give me 7 yuan?” (The right answer is 21 yuan), “What is the most and lowest amount of money I can give back to you? (The right answer is 30 and 0 yuan). Next, Classmate A asked whether the participants were ready. Afterward, a smiley face emoji (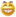) was displayed for the positive emoji group. The control group completed the trust game without any emojis. At the end of the experiment, the participants were asked, “Have you noticed the emoji in the WeChat frame?”

Finally, after completing the trust game, the participants who could not give the correct answer to the manipulation question or who did not observe the emoji were all excluded from analysis. the remaining participants were given money as compensation.

### Result

**FIGURE 2:**
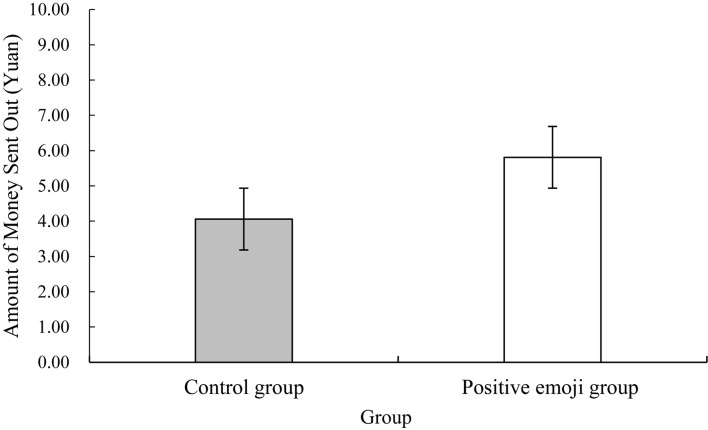
Differences between the amount of money sent by the positive emoji group and the control group.

Figure 2 demonstrates that the average values of the amounts given by the two groups of participants to the trustee are different. The amounts of money given by the experiment and control groups were 5.81 ± 2.76 and 4.06 ± 2.11 yuan, respectively. Given the amount of money as an index to measure the level of trust, an independent sample *t*-test was conducted. The results illustrate a significant difference in the amount of money sent out between the control and positive emoji groups, [*t*(60) = –2.79, *p* = 0.007, Cohen’s *d* = 0.71]. Compared with the control group, the positive emoji group received more money. This finding indicates that positive emojis can effectively improve the level of trust among trustors in the trust game.

## Experiment 2: Influence of Negative Emojis on Initial Online Trust Among College Students

### Participants

Experiment 2 randomly recruited a total of 74 college students in Beijing (37 males, 37 females; average age = 20.03, *SD* = 2.37). We conducted the same sensitivity power analysis (assuming *α* = 0.05, power = 0.8) in G^∗^Power 3.1 as experiment 1. The results showed that the effect size *d* we calculated based on our sample size was 0.66, which was at the medium level (0.50 < *d* < 0.80). All participants were randomly assigned to two groups, namely, the negative emoji and control groups, with 37 participants per group.

### Experimental Materials, Design, and Procedure

#### Negative Emoji

Using the method for Experiment 1, Experiment 2 employed the sad emoji (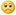), which is characterized by the downward corners of the mouth and downward slanting eyes.

#### Initial Online Trust

The material used was the same as in Experiment 1 except that the participants in the experiment group viewed a sad emoji.

The experiment adopted a single-factor (emoji: expressionless/sad) inter-participant design. The dependent variable was initial trust, whereas operation was defined as the amount of money that the participants submitted.

The experimental procedure was the same as that for Experiment 1.

### Result

**FIGURE 3:**
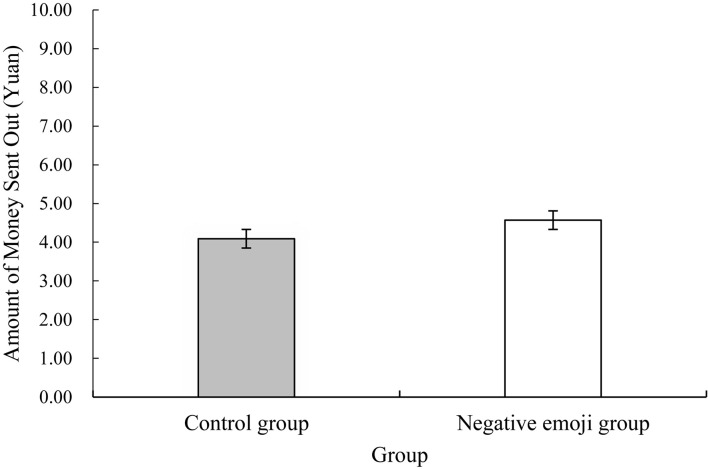
Differences between the amount of money sent by the negative emoji group and the control group.

Figure 3 indicates that the average value of the amount given to the trusted person by the two groups was slightly different (control group: 4.09 ± 3.26 yuan; experiment group: 4.57 ± 2.61 yuan). To verify whether the abovementioned differences reached significance, an independent sample *t*-test was conducted with the amount of money sent out by the two groups as the dependent variable. The results indicate that no significant difference exists in the initial level of trust between the control and negative emoji groups [*t*(72) = 0.69, *p* = 0.49, Cohen’s *d* = 0.16] and in the amount of money sent out. This finding illustrates that the negative emoji did not influence level of trust within the task.

## Joint Analysis of the Influence of Emoji Types on Initial Online Trust Among College Students

We conducted a joint analysis to examine the effects of emojis on initial level of trust among college students given the between-participant design of Experiments 1 and 2. Taking emojis as the independent variable and the amount of money sent out by the participants as the dependent variable, the study conducted one-way variance analysis (ANOVA). The three groups were composed of the control group from Experiment 1 (*n* = 31), the positive emoji group from Experiments 1 (*n* = 31) and the negative emoji group from Experiments 2 (*n* = 37), for a total of 99.

**FIGURE 4:**
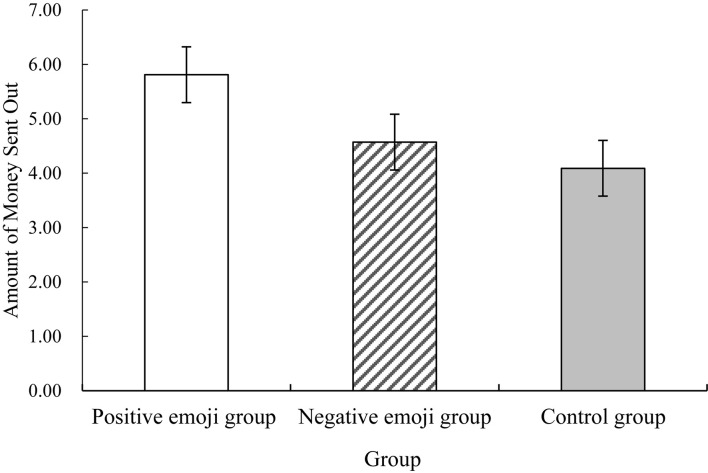
Differences between the amounts of money sent out by the control group and the positive and negative emoji groups.

The results demonstrated that different emojis have different effects on college students’ online trust [*F*(2,96) = 3.96, *p* = 0.02, η^2^ = 0.08]. After conducting a least significant difference test, we found that the positive emoji group received the largest amount of money [Figure 4; 5.81 ± 2.76 yuan (*M* ± *SD*)]; while the control group received the least amount of money (4.06 ± 2.11 yuan); and the negative emoji group received (4.57 ± 2.61 yuan). A significant difference was observed between the positive emoji and control groups (*p* = 0.008) and between the positive and negative emoji groups (*p* = 0.046) but not between the negative emoji and control groups (*p* = 0.41). The results confirm that positive instead of negative emojis can effectively improve the initial level of trust of the trustor in the investment game.

## Experiment 3: Influence of Emojis on Interpersonal Online Trust Among Acquaintances: Moderating Effects of Individual Trust Propensity

### Participants

The study randomly selected and recruited a total of 111 college students in Beijing (48 males, 63 females; average age = 18.87, *SD* = 1.434). In consistent with the previous 2 experiments, we conducted a sensitivity power analysis for the 2 (propensity to trust: high/low) × 3 (emojis: expressionless/smiley/sad) variance analysis based on online trust in experiment 3 (assuming α = 0.05, power = 0.8). The results showed that according to our sample size, the minimum effect size *f* we calculated for the main effects and interaction is 0.27, which is above the medium level (0.25 < *f* < 0.40). The participants were randomly assigned to control and positive and negative emoji groups with 37 participants per group.

### Experimental Materials

#### Individual Propensity to Trust

This construct was measured using single-question tests from the General Social Survey and the World Values Survey (Ben-Ner and Halldorsson, 2010), which are widely used in many disciplines. The item is, “Generally speaking, would you say that most people can be trusted, or that you can’t be too careful in dealing with people?” The item was rated using a binary response scale, where 0 = “You cannot be too careful when dealing with people” and 1 = “Most people can be trusted,” which represent low and high levels of propensity to trust, respectively.

### Experimental Design and Test Procedure

A 2 (propensity to trust: high/low) × 3 (emojis: expressionless/smiley/sad) inter-participant design was adopted with interpersonal trust as the dependent variable, whereas operation was defined as the amount of money given by the participant.

During the experiment, the individual propensity to trust was first measured using single-question tests from the General Social Survey and the World Values Survey. The participants then completed a trust game dialogue similar to that in Experiment 1. In contrast to the two previous experiments, the trustee was set as an acquaintance and denoted as Classmate F. To increase the sense of familiarity, the participants wrote down his/her names or codes. Other experimental procedures were the same as that of Experiment 1.

### Result

The emojis and propensity to trust were used as the independent variables, whereas the amount of money sent was the dependent variable. Two-factor ANOVA was conducted. The results indicated that the main effects of emojis [*F*(2,105) = 1.44, *p* = 0.24, η^2^ = 0.03] and the propensity to trust [*F* (1,105) = 2.25, *p* = 0.14, η^2^ = 0.02] were non-significant. However, the interaction between emojis and level of trust was significant [*F*(2,105) = 4.41, *p* = 0.01, η^2^ = 0.08].

**FIGURE 5:**
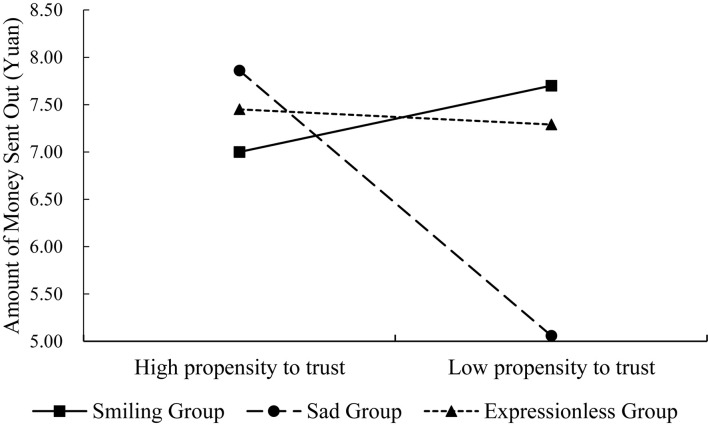
Interaction between emojis and propensity to trust in relation to interpersonal trust.

Further simple-effect analysis (Figure 5) illustrates that emojis exert a significant effect on interpersonal trust among acquaintances but only under the condition that individuals display a low level of propensity to trust [*F*(2,105) = 5.00, *p* = 0.008]. However, when the propensity to trust was high, emojis exerted no significant influence on network trust among acquaintances [*F*(2,105) = 0.50, *p* = 0.61]. Further analysis indicates that when the propensity to trust is low, the amount of money sent by the positive emoji group (*M* ± *SD* = 7.70 ± 2.23) is significantly higher than that of negative emoji group [*M* ± *SD* = 5.06 ± 2.93; *p* = 0.01, 95% CI = (0.51, 4.77)]. Furthermore, the amount of money sent by the negative emoji group is significantly less than that of the control group [*M* ± *SD* = 7.30 ± 2.62; *p* = 0.48, 95% CI = (–4.45, -0.51)]. Conversely, no differences were observed in the amount of money sent by the positive emoji and control groups [*p* = 0.95, 95% CI = (–1.69, 2.50)]. Therefore, participants with low levels of propensity to trust are more vulnerable to negative emotions in terms of interpersonal trust among acquaintances.

## Discussion

### Influence of Emojis on Online Initial Trust Among College Students

Emojis are visual representations of an individual’s point of view, experiences, feelings, or identity and can be used to express digital information on social media instead of or in combination with words (Willoughby and Liu, 2018). In 2014, the online edition of the Oxford Dictionary incorporated the word into an updated vocabulary, which renders it a universal language. Using emojis can easily add personality factors to text-based communication. Moreover, emojis are transmitted faster than input replies. Therefore, teenagers widely use emojis in online communication.

Research on emojis has only been conducted gradually in recent years, and it has mainly focused on the characteristics of emojis and their influence on other variables. On the one hand, various studies explore why emojis are widely used in computer-mediated text expression. An advantage of emojis is their ability to convey non-verbal information that may not be described using words. Moreover, their usage can strengthen emotional ties between the two parties of a communication and increase the sense of social intimacy by imitating actual facial expressions (Willoughby and Liu, 2018). On the other hand, other scholars discuss the influence of emojis on various economic phenomena as an expression of information. For example, they can be used as a method for directly evaluating the emotional connection between consumers and the food they buy and can be applied to consumer evaluation (Jaeger et al., 2017). The current study combines the abovementioned aspects by providing evidence of the influence of emojis on online trust among college students. As such, this study is highly innovative and is an important advancement of research in the field.

Previous studies and economic experiments have found that people hold high levels of trust expectations from strangers they met for the first time, unless evidence of untrustworthy behavior exists (Cañadas et al., 2015). This study further confirmed that emojis, as an important social clue, can improve the initial level of trust between strangers. On one hand, this result is due to the presence of facial clues, which is consistent with those of previous studies that suggest that a figure composed of dots that resemble a human face can promote trust. This type of facial clue can provide psychological hints, enhance prosocial behaviors, and improve the initial level of trust among participants (Xin et al., 2016). On the other hand, developments in online trust are also due to the effect of emojis on network telepresence (Dai et al., 2018). Using emojis in online chats enables online communication to feel similar to face-to-face communication, which imbues participants with a sense of reality and familiarity. In addition, it can decrease the psychological distance between participants and improve initial trust.

### Influencing Mechanism of Different Emojis on Initial Online Trust Among College Students

The results for Experiments 1 and 2 indicated that positive emoji, as represented by the smiley emoji, can effectively improve the initial level of trust within the network of a trustor, whereas the opposite is not true for negative emoji represented by sad emoji. This notion is consistent with that of a previous study on positive emotions (Ganster et al., 2012). Moreover, negative expressions exerted the same influence on trust as neutral expressions (Cañadas et al., 2015). Evidently, it is the emotion conveyed and caused by emojis influence the level of initial online trust instead of the mere appearance of a given emoji.

Positive emojis (i.e., smileys), can improve the initial levels of trust within a network, which is closely related to the projection and empathy of the personality characteristics of users. According to the projection principle (Marengo et al., 2017), emojis not only reflect a user’s personality traits but also dynamically constructs and shapes its image (Bai et al., 2019). In Experiment 1, the positive emoji appeared immediately after the description of the rules of the trust game, which means that trustee conveys to trustor the propensity to be intimate and cooperative. Thus, this notion stimulates positive emotions directed toward the trusted person. As such, these findings are consistent with those of previous studies, that is, positive emojis improve the levels of trust displayed toward the trusted person (Lount, 2010). Previous analysis of emotions related to emojis found that the majority of emojis are positive, given that the most popular expressions carry more emotional information (Novak et al., 2015).

Negative emojis (i.e., sad face) may exert no influence on online initial trust because the trustor explained the motive of the trustee when using this expression. According to previous studies, among the seven motivations of individuals for using emojis, the most important one is the expression of different emotions, especially in relation to strengthening positive emotions. In general, the evident context of the expression of negative emotions is the elimination of ambiguity (Hu et al., 2017). In the current study, however, the emotion conveyed by the sad emoji, which immediately followed the description of the game rules, is ambiguous. This notion can be understood as a hint that a formal experiment is required immediately or that an argument should be raised about the complexity of the rules. It may even represent the intention to use negative expressions to enhance intimacy on both sides of the communication, as posited in previous studies (Fernández-Gavilanes et al., 2018). Therefore, the results of using negative emoji differ from those of using positive emojis in that the trustor have dynamic opportunities to understand what the negative emoji really mean. It might lead to the dispersion of the changing trend within a trust game. In other words, the trust level in this group is similar to that of the group that does not use an emoji. In addition, previous studies found high levels of propensity to trust toward strangers (Cañadas et al., 2015), which is another reason why ambiguous negative emojis cannot be used as sufficient evidence to alter the trust tendencies of trustors.

### Influence of Emojis on Online Interpersonal Trust Among Acquaintance

Experiment 3 reveals that different emojis exerted no significant influence on interpersonal online trust among the acquaintances of college students. However, the propensity to trust plays a moderating role in this relationship.

On the one hand, the current study found that different emojis and levels of trust exerted no significant influence on interpersonal online trust among college students and their acquaintances. This finding is consistent with those of previous research on the relationship between trust (as measured by trust games) and emotions (Dunn and Schweitzer, 2005). When two participants of the game are familiar with each other, different emojis, as a medium for conveying emotions, also exert no effect. According to famous sociologist Luhmann (1979), trust is a mechanism used to reduce the complexity of social interactions. Furthermore, it can surpass the existing information to summarize several behavioral expectations, which, thus, compensates for the required information through a sense of security. However, interactions between acquaintances are based on trust. Therefore, neither an emoji’s expression (Xin et al., 2016) as a subtle social clue nor an individual’s propensity to trust will influence levels of trust.

On the other hand, Experiment 3 revealed that individual propensity to trust played a moderate role in the relationship between the use of emojis and online trust. Only when the individual propensity to trust is low, emoji could play a key role in online trust between acquaintances. This finding is consistent with those of previous studies and proves, once again, the fragility of new relationships within contemporary social networks and the important role of individual levels of trust (Fischer and Herbert, 2021). Individuals with high levels of propensity to trust are prone to make positive judgments regarding the abilities, goodwill, integrity, and other characteristics of other people. As such, these individuals tend to participate in more prosocial behaviors, such as providing help and sharing relevant benefits with others, thereby maintaining social order and economic prosperity (Ben-Ner and Halldorsson, 2010; Cao et al., 2020). Therefore, the role of an individual’s propensity to trust can surpass the role of emojis in terms of emotional cues. On the contrary, when an individual’s propensity to trust is low, he/she is usually cautious in establishing trusting relationships with others and are more sensitive to subtle social clues. This notion is especially true in the face of a network background, where such individuals tend to trust their acquaintances as much as they do strangers. The expression of a smiley face provides a clue on positive emotions, whereas the sad-faced emoji represents negative emotions, which may arouse different emotional experiences. In turn, this stimulation may influence levels of online trust (Wei et al., 2020).

### Limitations

This study has certain limitations. First, the individual propensity to trust (Experiment 3) was measured using a single-question test widely used in many studies, however, there also many other measures for multiple dimensions of trust (Ben-Ner and Halldorsson, 2010). Thus, future research should use other methods to measure this aspect to further explore the impact of emojis on trust. Second, the survey was conducted only on college students. Future studies should further test whether the conclusion found in this study are applicable to other groups, such as adolescents or young adults. Third, in terms of the experiment materials, we selected only one type of emoji that best represent positive and negative facial expressions, but there are hundreds of emojis (Novak et al., 2015).

Thus, future studies should consider various types of emojis to investigate the effects of other emojis on trust. Finally, we investigated only the role of individual propensity to trust in the relationship between emojis and online trust. However, previous research found that personality traits, such as extroversion, also influence individual trust decisions (Cao et al., 2020). Therefore, investigating the influence of additional variables, such as extraversion, on the abovementioned relationship may be an interesting avenue for future research.

## Conclusion

In conclusion, this study analyzed the effect of emojis on online trust of college students and the boundary of its role through 3 experiments. Specifically, emojis that convey positive emotions, such as a smiley face, could increase initial online trust, whereas emojis that convey negative emotions, like sad face, do not influence initial online trust unfamiliar college students. Furthermore, college students with low levels of trust propensity are more vulnerable to negative emotions in the trust game. This study not only has important theoretical implications for research in the area of trust, but also provides important practical inspiration for educators to intervene in the level of interpersonal trust among college students.

## Data Availability Statement

The raw data supporting the conclusions of this article will be made available by the authors, without undue reservation.

## Ethics Statement

All procedures performed in the studies involving human participants were conducted in accordance with the Ethical Standards of the Administration Committee of Psychological Research in the Central University of Finance and Economics and with the 1964 Helsinki Declaration and its later amendments or comparable ethical standards. Informed consent was obtained from all participants.

## Author Contributions

MZ conceived and designed the study. SD and YL conducted the study. HL analyzed the data. MZ, YZ, and CQ drafted the manuscript. All authors contributed to the article and approved the final version.

## Conflict of Interest

The authors declare that the research was conducted in the absence of any commercial or financial relationships that could be construed as a potential conflict of interest.

## Publisher’s Note

All claims expressed in this article are solely those of the authors and do not necessarily represent those of their affiliated organizations, or those of the publisher, the editors and the reviewers. Any product that may be evaluated in this article, or claim that may be made by its manufacturer, is not guaranteed or endorsed by the publisher.
